# Eupatilin Impacts on the Progression of Colon Cancer by Mitochondria Dysfunction and Oxidative Stress

**DOI:** 10.3390/antiox10060957

**Published:** 2021-06-15

**Authors:** Minkyeong Lee, Changwon Yang, Gwonhwa Song, Whasun Lim

**Affiliations:** 1 Department of Food and Nutrition, College of Science and Technology, Kookmin University, Seoul 02707, Korea; m2019546@kookmin.ac.kr; 2 Institute of Animal Molecular Biotechnology and Department of Biotechnology, College of Life Sciences and Biotechnology, Korea University, Seoul 02841, Korea; ycw117@korea.ac.kr

**Keywords:** eupatilin, colon cancer, apoptosis, oxidative stress, drug resistance

## Abstract

Colon cancer is one of the most frequently diagnosed cancer types. Some colon cancer cases resist standard anticancer drugs. Therefore, many studies have focused on developing therapeutic supplements using natural products with low side effects and broad physiological activity. Eupatilin is a flavonoid that is mainly extracted from artemisia and promotes apoptosis in numerous cancer types. However, since the current understanding of its physiological mechanisms on colon cancer cells is insufficient, we investigated how eupatilin affects the growth of two colon cancer cell lines, namely HCT116 and HT29. Our results showed that eupatilin inhibits cell viability and induces apoptosis accompanied by mitochondrial depolarization. It also induces oxidative stress in colon cancer cells and regulates the expression of proteins involved in the endoplasmic reticulum stress and autophagic process. Moreover, eupatilin may target the PI3K/AKT and mitogen-activated protein kinase (MAPK) signaling pathways in colon cancer cells. It also prevents colon cancer cell invasion. Furthermore, eupatilin has a synergistic effect with 5-fluorouracil (5-FU; a standard anticancer drug) on 5-FU-resistant HCT116 cells. These results suggest that eupatilin can be developed as an adjuvant to enhance traditional anticancer drugs in colon cancer.

## 1. Introduction

In 2020, colon cancer ranked third in new cancer cases and cancer-related deaths in both men and women in the United States [1]. The high mortality rate of colon cancer comes from the development of chemoresistance and the high rate of metastasis [2]. Almost 50% of colon cancer cases have mutations in the tumor-suppressor P53 protein, which promotes cell proliferation and invasion, chemoresistance [3]. 5-fluorouracil (5-FU) is the recommended standard drug in clinical research, and it can be used alone or in combination with irinotecan and oxaliplatin [4]. Despite improvements in treatment options, resistance to traditional drugs remains frequent, and novel therapeutic adjuvants need to be developed. [5].

Flavonoids are polyphenols commonly found in edible plants. They are biologically active with low side effects, so their development potential as therapeutic adjuvants in various diseases has drawn attention. Artemisia, which contains eupatilin, is a plant that is widely used in oriental medicine and is well known for its anti-inflammatory and antioxidant activity [6,7]. Numerous studies have reported that the apoptosis-inducing effect of eupatilin is effective against gastric, prostate, cervical, esophageal, and endometrial cancer [8,9,10,11,12,13]. These studies have revealed that eupatilin can regulate various physiological mechanisms associated with proliferation, cell cycle, and invasion in cancer cells. In addition, our previous study using a transgenic zebrafish model revealed that eupatilin has an anti-angiogenic effect [14]. Moreover, a synthetic derivative of eupatilin prevents dextran sulfate sodium-induced colitis and inflammation-related colon carcinogenesis [15]. However, knowing whether and how eupatilin induces apoptosis in colon cancer cells requires further investigation. Therefore, in this study, we treated HCT116 and HT29 cells with eupatilin to estimate its effects on cell viability, cell cycle, and invasiveness. We also revealed that eupatilin regulates oxidative stress, mitochondrial permeabilization, and cancer cell growth- and survival-related signaling pathways.

## 2. Materials and Methods

### 2.1. Chemicals

Eupatilin was obtained from Syngene International Ltd. (Karnataka Bengaluru, India) and LY294002 from Cell Signaling Technology (Danvers, MA, USA). U0126, SP600125, and SB203580 were purchased from Enzo Life Sciences (Farmingdale, NY, USA). 5-FU and irinotecan were acquired from Sigma (St.Louis, MO, USA). Table 1 lists the antibodies used in Western blot analysis.

### 2.2. Cell Culture

HCT116, HT29, and CCD-18Co cells were purchased from the Korean Cell Line Bank (Seoul, Korea). To establish colon cancer cells resistant to 5-FU (5FUR), HCT116 was cultured for at least 6 months with progressively increasing 5-FU concentrations starting at 0.5 μM. In our prior experiment, the half maximal inhibitory concentration (IC_50_) of cell viability to 5-FU of 5-FUR HCT116 cells was 16.1 μM, whereas in parental HCT116 cells, it was 1.9 μM.

### 2.3. Proliferation Assay and Cell Viability Test

To assess proliferation, the cells were incubated with a bromodeoxyuridine solution for 2 h and again for 1 h and 30 min. The relative absorbance value at the 370/492 nm wavelength was estimated using a microplate reader and quantified with at least three microplate wells. Cell viability changes on the HCT116 and HT29 cells were monitored in response to eupatilin for 48 h using an 3-(4,5-dimethylthiazol-2-yl)-2,5-diphenyltetrazolium bromide (MTT) labeling reagent (Roche, Basel, Switzerland) according to manufacturer’s instructions.

### 2.4. Spheroid Culture

To form spheroids for colon cancer cells, 3000 cells were seeded onto the lid of the culture dish with eupatilin treatment [16]. HCT116 and HT29 cells were cultured for 3 and 4 days, respectively, with 50 μM of eupatilin. The morphological changes were observed with Leica’s DM3000 microscope (Wetzlar, Germany). The total area for each image was quantified with the ImageJ software (NIH, Bethesda, MD, USA) (http://rsb.info.nih.gov/ij/docs/index.html (accessed on 10 April 2021)). The ReViSP software (MathWorks, Natick, MA, USA) (https://sourceforge.net/projects/revisp/ (accessed on 10 April 2021)) was used to measure the density of spheroids. Briefly, the mask image obtained by the ImageJ software was converted to a spherical 3D image by the ReViSP software, and the density was quantified based on the automatically calculated volume values. At least three independent spheroid images were quantified. 

### 2.5. Annexin V and Propidium Iodide Staining

Annexin V and propidium iodide (PI) were used to detect apoptotic HCT 116 and HT29 cells treated with eupatilin. The eupatilin-treated cells were harvested and stained with both dyes for 15 min, and fluorescence intensity was quantified with a FACSCalibur flow cytometer (BD Biosciences, Franklin Lakes, NJ, USA).

### 2.6. Terminal Deoxynucleotidyl Transferase 2’-Deoxyuridine-5’-Triphosphate (dUTP) Nick end Labeling (TUNEL) Assay

The cells were air dried and fixed with 4% paraformaldehyde in phosphate buffered saline (PBS) for 1 h at room temperature. The cells were briefly rinsed with PBS and permeabilized with 0.1% Triton X-100 in 0.1% sodium citrate for 2 min on ice. Then, the cells were subjected to a TUNEL staining mixture using the In Situ Cell Death Detection kit, TMR red (Roche) for 1 h at 37 °C in the dark. Cells then were washed with PBS and overlaid with 4′,6-diamidino-2-phenylindole (DAPI). Fluorescence was detected using an LSM710 (Carl Zeiss, Oberkochen, Germany) confocal microscope.

### 2.7. JC-1 Staining

JC-1 dye in a mitochondrial staining kit (Sigma) was used to detect the mitochondrial membrane potential of HCT116 and HT29 cells treated with eupatilin for 48 h. The eupatilin-treated cells were harvested and stained with JC-1 for 20 min, and fluorescence intensity was quantified with a FACSCalibur flow cytometer (BD Biosciences).

### 2.8. Western Blot Analysis

Protein extraction, denaturation, and Western blot methods were described in a previous work [17]. Western blots were conducted on proteins extracted from colon cancer cells treated with eupatilin for 24 h.

### 2.9. Cell Cycle Analysis

The cycle distribution in colon cancer cells was analyzed through PI staining with RNase A (100 μg/mL) treatment, as described in a previous study [14].

### 2.10. Reactive Oxygen Species (ROS) Analysis

To estimate ROS production in HCT116 and HT29 cells, they were treated with eupatilin for 2 h and then with 2′,7′-dichlorofluorescein diacetate for 30 min. Fluorescence intensity was measured with a FACSCalibur flow cytometer.

### 2.11. Transwell Invasion Assay and Migration Assay

Cell invasion was analyzed using 8-μm pore Transwell inserts (Cat No: 3422, Corning, Inc., New York, NY, USA) coated with a Matrigel^®^ growth factor reduced basement membrane matrix (Cat No: 356230, Corning, Inc., Corning, NY, USA) for 2 h at 37 °C. Cells in serum-free medium containing 50 μM of eupatilin were plated in the upper chamber, while a medium containing 10% FBS was added to the lower wells. After cells were incubated for 16 h at 37 °C in a CO_2_ incubator, cells that had not invaded were removed with a cotton swab. For the evaluation of cells that invaded onto the lower surface, inserts were fixed in methanol for 10 min. The Transwell membranes were then air-dried and stained using hematoxylin (Sigma) for 30 min. The hematoxylin-stained cells were counted using Leica’s DM3000 microscope. Cell migration was also analyzed using culture-insert 2-well in µ-dish 35 mm (Cat No: 81176, ibidi GmbH, Munich, Germany) according to the manufacturer’s instructions. The degree of migration to the cell-free gap during 24 h of treatment with 50 μM of eupatilin was observed through Leica’s DM3000 microscope. Based on the gap closure, the migration of cells was quantified.

### 2.12. Statistics

The statistical significance of all experiments was estimated using the SAS program (9.4 version, Cary, NC, USA). All experiments were repeated at least three times. A probability value of *p* < 0.05 was considered statistically significant.

## 3. Results

### 3.1. Eupatilin Inhibits Colon Cancer Cell Growth

**Figure 1:**
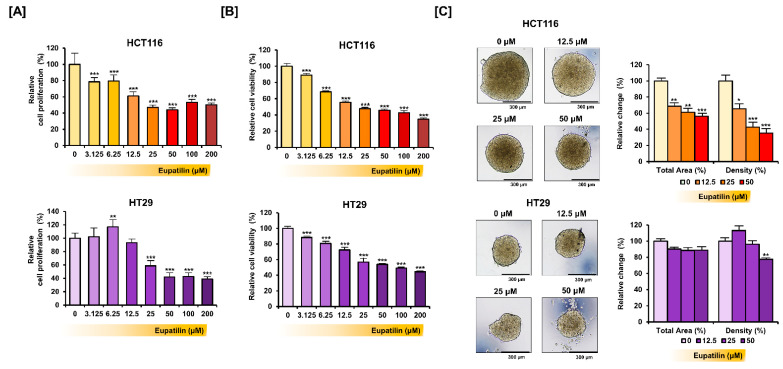
Eupatilin inhibits colon cancer cell growth. (**A**) Dose-dependent effects of eupatilin on HCT116 and HT29 cell proliferation. (**B**) Dose-dependent effects of eupatilin on HCT116 and HT29 cell viability. (**C**) Bright field images showing the changes in spheroid morphology following eupatilin treatment. The total area and density of the spheroid were calculated using the ImageJ software. Asterisks indicate a significant change after eupatilin treatment (*** *p* < 0.001, ** *p* < 0.01 and * *p* < 0.05).

Cell proliferation changes were first analyzed after treating HCT116 and HT29 cells with increasing eupatilin doses (Figure 1A). Cell proliferation decreased by more than 50% at concentrations above 25 μM in HCT116 cells and above 50 μM in HT29 cells. Under the same conditions, cell viability decreased by more than 50% at 25 μM or more (*p* < 0.001) in HCT116 cells and 100 μM or more (*p* < 0.001) in HT29 cells (Figure 1B). Next, we induced spheroid formation in HCT116 and HT29 cells through the hanging drop method with the dose-dependent treatment of eupatilin (Figure 1C). In HCT116 cells, eupatilin decreased the total area and density of spheroids in a dose-dependent manner. However, in HT29 cells, eupatilin did not significantly decrease the total area of the spheroid and only significantly reduced the density by treatment at a concentration of 50 μM (*p* < 0.01).

### 3.2. Eupatilin Induces Apoptotic Processes in Colon Cancer Cells

**Figure 2:**
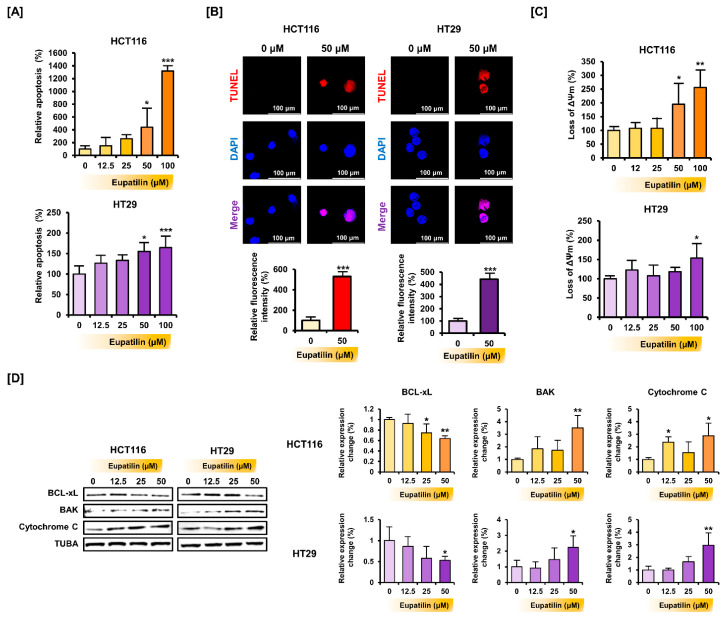
Eupatilin induces apoptosis in colon cancer cells. (**A**) Apoptosis detection in eupatilin-treated HCT116 and HT29 cells. Annexin V and propidium iodide (PI) fluorescence values were estimated by flow cytometry. (**B**) TUNEL fluorescence immunocytochemistry field showed apoptotic cells (red), and the cells were counter-stained with DAPI (blue) (**C**) Mitochondrial membrane potential alteration in eupatilin-treated HCT116 and HT29 cells, detected by flow cytometry. (**D**) Analysis of BCLXL, BAK, and cytochrome c expression after treatment with various eupatilin concentrations for 24 h. Immunoblots were quantitated to calculate normalized values relative to alpha-tubulin (TUBA). Asterisks indicate a significant change after eupatilin treatment (*** *p* < 0.001, ** *p* < 0.01, and * *p* < 0.05).

Whether treating colon cancer cells with eupatilin for 48 h induced apoptosis was then investigated (Figure 2A). In HCT116 cells, 50 and 100 µM of eupatilin significantly increased apoptosis (4.4-fold (*p* < 0.05) and 13.2-fold (*p* < 0.001), respectively). Moreover, eupatilin significantly promoted apoptosis in HT29 cells at 50 μM (1.6-fold; *p* < 0.05) and 100 μM (1.7-fold; *p* < 0.001). Based on the change in the characteristics of HCT116 and HT29 cells following the dose-dependent treatment of eupatilin, 50 μM was set as the optimal treatment concentration to analyze the apoptotic effect after treatment with a single eupatilin concentration. To observe the process of apoptosis by microscopic imaging, a TUNEL assay showing DNA fragmentation was performed (Figure 2B). As a result, eupatilin increased the frequency of DNA fragmentation indicated by red fluorescence in HCT116 and HT29 cells. Next, staining with JC-1 was done to determine whether eupatilin-induced apoptosis mediates mitochondrial dysfunction (Figure 2C). Eupatilin significantly decreased mitochondrial membrane potential at 50 μM (2.0-fold; *p* < 0.05) and 100 μM (2.6-fold; *p* < 0.01) in HCT116 cells and 100 μM (1.5-fold; *p* < 0.05) in HT29 cells. Next, the cells were treated with various eupatilin concentrations for 24 h, whole proteins were extracted, and the expression of apoptosis-related proteins was analyzed by Western blot (Figure 2D). Eupatilin suppressed BCL-xL expression, but it increased BAK and cytochrome c expression.

### 3.3. Eupatilin Regulates the Cell Cycle Phases and Promotes ROS Production in Colon Cancer

**Figure 3:**
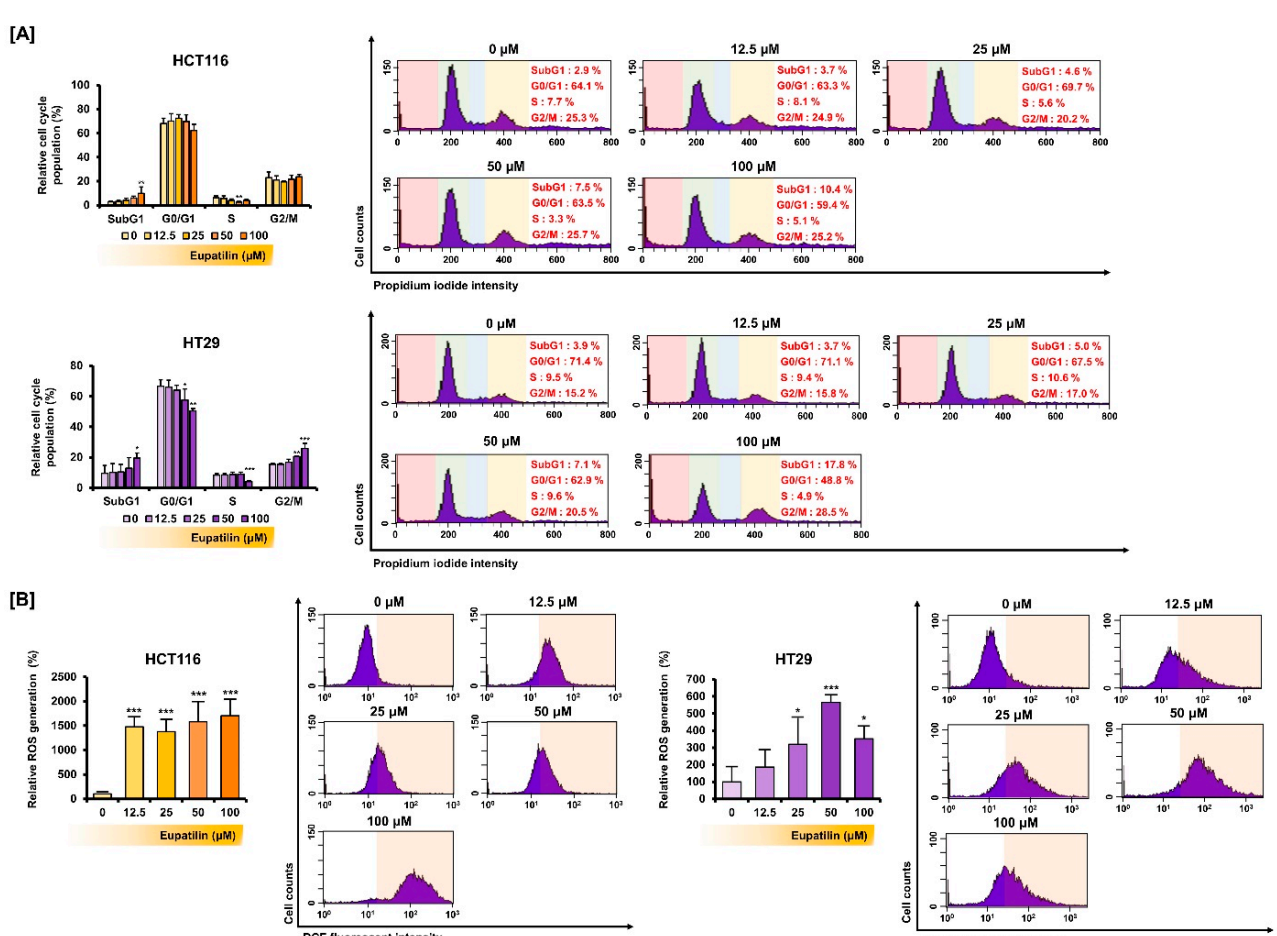
Effect of eupatilin on cell cycle and ROS production in colon cancer cells. (**A**) Cell cycle distribution was confirmed by PI staining. (**B**) Eupatilin-induced intracellular ROS production estimated by flowcytometric detection. The number of DCF green fluorescence-labeled cells represents the relative quantities of intracellular hydrogen peroxide in eupatilin-treated cells. Asterisks indicate a significant change after eupatilin treatment (*** *p* < 0.001, ** *p* < 0.01, and * *p* < 0.05).

Next, how treatment with various eupatilin concentrations for 48 h affected the colon cancer cell cycle was investigated (Figure 3A). At 100 μM, eupatilin significantly increased the SubG1 phase population, suggesting progression to cell death. On the other hand, 100 µM of eupatilin decreased the S phase population. Then, we estimated the changes in ROS production, a representative mechanism that induces cancer cell death, through 2′,7′-dichlorofluorescin diacetate (DCFH-DA) treatment (Figure 3B). Peroxide converts DCFH-DA to fluorescent 2′,7′-dichlorofluorescein (DCF). Eupatilin increased DCF fluorescence intensity in HCT116 and HT29 cells, suggesting excessive ROS production. Treatment with 50 μM of eupatilin increased ROS production by 15.8-fold (*p* < 0.001) in HCT116 cells and 5.6-fold (*p* < 0.001) in HT29 cells.

### 3.4. Eupatilin Regulates the Proteins Involved in the PI3K/AKT and MAPK Pathways, Endoplasmic Reticulum (ER) Stress, and Autophagy in Colon Cancer Cells

**Figure 4:**
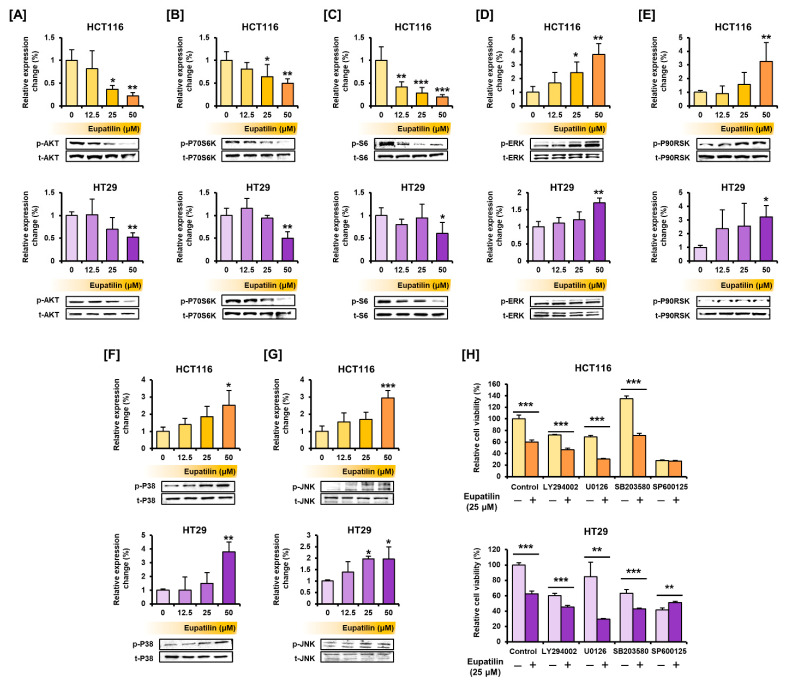
Regulation of PI3K/AKT and MAPK signaling pathways by eupatilin in colon cancer cells. (**A**–**G**) Phosphorylated AKT (**A**), P70S6K (**B**), S6 (**C**), ERK (**D**), P90RSK (**E**), P38 (**F**), and JNK (**G**). Immunoblots were quantitated to calculate the normalized values by estimation of expressed levels from phosphorylated proteins relative to total proteins. (**H**) Effects of eupatilin and selective inhibitors for AKT (LY294002), ERK (U0126), P38 (SB203580), and JNK (SP600125) on HCT116 and HT29 cell viability. Data are presented as a percentage relative to control (100%). Asterisks indicate a significant change after eupatilin treatment (*** *p* < 0.001, ** *p* < 0.01, and * *p* < 0.05).

The phosphorylation of proteins of the representative signaling pathways involved in cell growth and survival was investigated by Western blot. In HCT116 and HT29 cells, eupatilin inhibited the phosphorylation of AKT and its downstream proteins P70 ribosomal protein S6 kinase (P70S6K) and S6, which are part of the PI3K/AKT signaling pathway (Figure 4A–C). In contrast, eupatilin induced the phosphorylation of ERK in HCT116 and HT29 cells, as well as of the P90 ribosomal S6 kinase (P90RSK), a downstream protein (Figure 4D,E). Eupatilin also increased the phosphorylation of P38 and the c-Jun N-terminal kinase (JNK) (Figure 4F,G). Next, cell viability was confirmed after co-treatment with LY294002, U0126, SB203580, and SP600125, which are selective inhibitors of AKT, ERK, P38, and JNK, respectively (Figure 4H). Co-treatment with LY294002, U0126, and SB203580 in HCT116 and HT29 cells further reduced the cell viability suppressed by eupatilin. These results suggest that eupatilin activates signaling pathways that regulate the cell viability of colon cancer cells.

**Figure 5:**
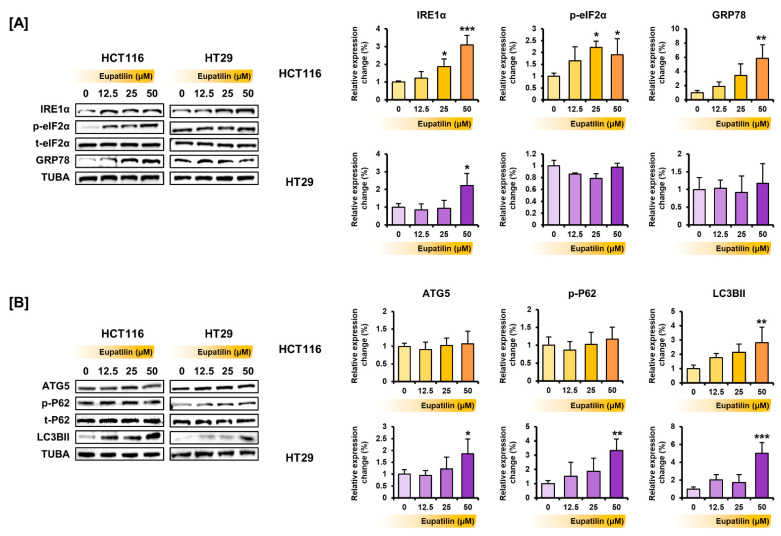
Modulatory effect of eupatilin on protein expression related to ER stress and autophagy in colon cancer cells. (**A**) IRE1α, phosphorylated eIF2α, total eIF2α, and GRP78 expression levels in response to various eupatilin for 24 h. (**B**) ATG, phosphorylated P62, total P62, and LC3BII expression levels in response to various eupatilin concentrations for 24 h. Immunoblots were quantitated to calculate normalized values relative to TUBA. Asterisks indicate a significant change after eupatilin treatment (*** *p* < 0.001, ** *p* < 0.01, and * *p* < 0.05).

Next, whether eupatilin could regulate endoplasmic reticulum (ER) stress-related protein expression was investigated. After treating the colon cancer cells with various eupatilin concentrations, the proteins were extracted, and Western blot confirmed that eupatilin increases the expression of ER stress-related proteins (Figure 5A). Eupatilin increased the expression of inositol-requiring enzyme alpha (IRE1α), phosphorylated eukaryotic initiation factor 2 alpha (eIF2α), and glucose-regulated protein 78 (GRP78) in HCT116 cells and only of IRE1α in HT29 cells. How eupatilin affected autophagy-related proteins was also investigated. Eupatilin increased the expression of light chain 3B type II (LC3BII) in HCT116 cells and of autophagy-related 5 (ATG5), P62, and LC3BII in HT29 cells (Figure 5B).

### 3.5. Eupatilin Inhibits Invasion and Migration in Colon Cancer Cells

**Figure 6:**
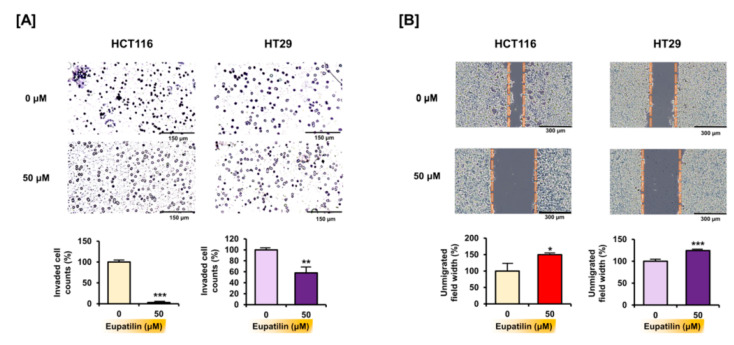
Eupatilin inhibits invasion and migration in colon cancer cells. (**A**) Invasive capacities were measured using the Transwell migration assay. Eupatilin significantly decreased invasion. (**B**) Migration of HCT116 and HT29 cells in response to eupatilin calculated based on gap distance. Asterisks indicate a significant change after eupatilin treatment (*** *p* < 0.001, ** *p* < 0.01, and * *p* < 0.05).

Through Transwell migration assay analysis, it was found that eupatilin can regulate cell invasion, which is essential for cancer cell growth and metastasis. Treatment with 50 μM of eupatilin significantly reduced the number of HCT116 (by 97.2%; *p* < 0.001) and HT29 (by 42.0%; *p* < 0.01) cells that passed through the membrane (Figure 6A). Moreover, eupatilin inhibited the proportion of HCT116 and HT29 cells migrating to the empty space, as proven by the large interspace (Figure 6B).

### 3.6. Eupatilin Has a Synergistic Effect with Standard Anticancer Drugs on Colon Cancer Cells

**Figure 7:**
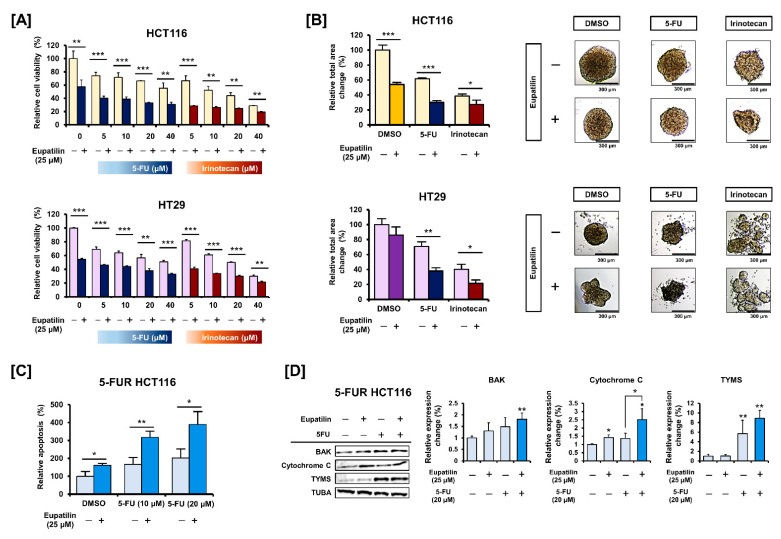
Synergistic effect of eupatilin combined with standard anticancer drugs. (**A**) Effects of eupatilin combined with 5-FU or irinotecan on HCT116 and HT29 cell viability. (**B**) Bright field images showing the changes in spheroid morphology following eupatilin treatment combined with 5-FU or irinotecan. The total area and density of the spheroid were calculated using the ImageJ software. (**C**) Flow cytometric detection of apoptosis in 5-FUR HCT116 cells in response to 5-FU and eupatilin combined treatment using Annexin V and PI staining. (**D**) BAK, cytochrome c, and TYMS expression levels in response to eupatilin and 5-FU combined treatment for 24 h in 5FUR HCT116 cells. Asterisks indicate a significant change after eupatilin treatment (*** *p* < 0.001, ** *p* < 0.01, and * *p* < 0.05).

Eupatilin’s effect in combination with the standard anticancer drugs 5-FU and irinotecan was investigated. HCT116 and HT29 cell viability treated with various 5-FU and irinotecan concentrations (5, 10, 20, and 40 μM) was assessed in the presence or absence of eupatilin (Figure 7A). Eupatilin further reduced cell viability when combined with irinotecan and 5-FU at all concentrations. Microscopic observations confirmed that co-treatment with eupatilin and 5-FU or irinotecan might further impair spheroid formation (Figure 7B). HCT116 cells were then treated with 5-FU over a long period, and relatively 5-FU-resistant (5-FUR) cells were obtained. Eupatilin significantly increased the 5-FU-induced apoptosis in 5-FUR HCT116 cells (Figure 7C). We confirmed that 5-FUR HCT116 cells expressed significantly more thymidylate synthase (TYMS), a target protein of 5-FU (Figure 7D). Furthermore, 5-FU alone did not affect BAK and cytochrome c expression in 5-FUR HCT116 cells, while the combination of 5-FU and eupatilin increased it. These results imply that eupatilin supports standard anticancer drugs by mediating synergistic effects in colon cancer cells.

### 3.7. Eupatilin Does Not Cause Changes in the Properties of Normal Colon Cells

**Figure 8:**
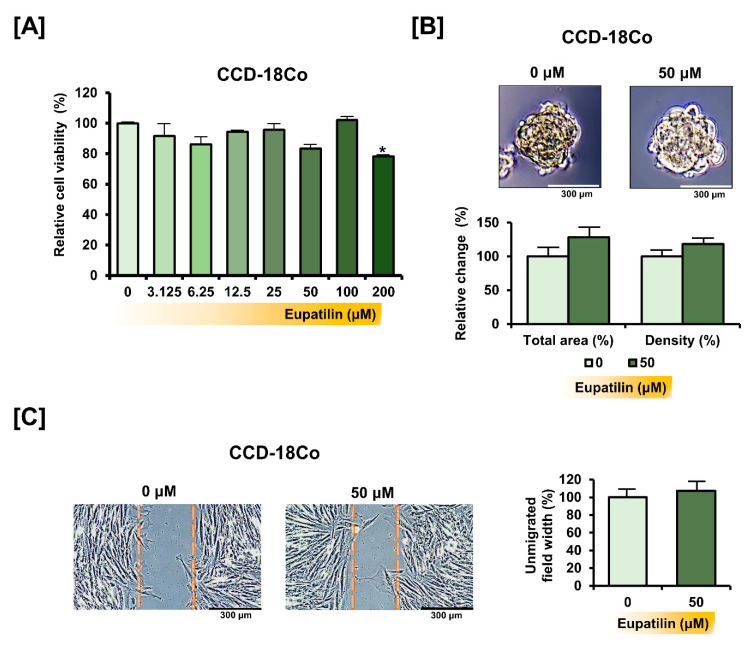
Effect of eupatilin in normal colon cancer cells. (**A**) Dose-dependent effects of eupatilin on CCD-18Co cell viability (**B**) Bright field images showing the changes in spheroid morphology following eupatilin treatment. The total area and density of the spheroid were calculated using the ImageJ software. (**C**) Migration of CCD-18Co cells in response to eupatilin calculated based on gap distance. Asterisks indicate a significant change after eupatilin treatment (* *p* < 0.05).

We dose-dependently treated the CCD-18Co cell line with eupatilin to determine if eupatilin can cause toxicity to normal colon cells. Eupatilin did not have a significant effect on the viability of CCD-18Co cells, except for the 200 μM treatment (Figure 8A). Even on the total area and density of the spheroids of CCD-18Co, 50 μM of eupatilin did not have a significant effect (Figure 8B). In addition, eupatilin did not significantly affect the migration of CCD-18Co cells (Figure 8C). These results suggest that eupatilin has a minor effect on cellular properties in normal colon cells, unlike in colon cancer cells.

## 4. Discussion

We found that the physiological regulation of eupatilin in colon cancer cells is similar to that of other cancer cell types. For instance, eupatilin induces apoptosis by mediating cytochrome c release from mitochondria in leukemia cells [12]. Moreover, in gastric cancer cells, eupatilin induces apoptosis by regulating apoptotic proteins such as BAX and BCL2 and inducing mitochondrial depolarization [8]. Eupatilin also induces apoptosis by mediating mitochondrial membrane depolarization in cervical cancer cells [18]. Similarly, we found that eupatilin can regulate the expression of the mitochondrial proteins BAK, BCL-xL, and cytochrome c, which are involved in apoptotic processes in colon cancer cells, as well as in mitochondrial depolarization. Microscopic imaging, which showed that eupatilin induced DNA fragmentation, indicated the apoptosis-inducing effect of eupatilin in colon cancer cells. When the mitochondrial membrane potential decreases, mitochondria release cytochrome c and ROS [19]. Increasing oxidative stress levels within cancer cells comprise an important therapeutic strategy, because it makes cells vulnerable to further ROS increase by therapeutic compounds [20]. It has been speculated that normal colon cancer cells, which have lower levels of oxidative stress compared to cancer cells, are less sensitive to compounds that produce ROS. In this study, eupatilin did not significantly affect the viability or migration of CCD-18Co cells, a normal colon cancer cell line. These results suggest that eupatilin may induce oxidative stress-mediated apoptosis specific to colon cancer cells, although further research is needed. Excessive intracellular ROS production activates signaling pathways that induce oxidative stress and promote cell cycle arrest and apoptosis [21]. Our results suggest that eupatilin can regulate the cell cycle and induce apoptosis by mediating excessive ROS production in colon cancer cells. In HCT116 cells, eupatilin clearly increased the SubG1 phase population. In HT29 cells, eupatilin induced cell arrest in the G2/M phase and increased the SubG1 phase population at a high concentration. The difference in cell cycle regulation between two cell lines suggests that eupatilin mediates P53, which requires confirmation. Forming spheroids is a useful model for confirming 3D morphological changes in vitro in cancer research. Eupatilin was found to inhibit the spheroid size of colon cancer cells, suggesting that eupatilin may inhibit colon cancer growth [22].

Moreover, in this study, it was verified that eupatilin can cause synergistic effects in colon cancer cells when co-treated with traditional anticancer drugs for colon cancer such as 5-FU or irinotecan. In particular, the combination treatment of eupatilin and 5-FU increased the expression of BAK and cytochrome c even more than the respective treatments alone, leading to an increase in apoptosis of colon cancer cells. The TYMS is a target protein for 5-FU, and the inhibition of TYMS is considered a therapeutic strategy to increase the sensitivity of 5-FU. However, in this study, eupatilin did not have a significant effect on TYMS expression in colon cancer cells. These results suggest that eupatilin will cause a synergistic effect with 5-FU in a TYMS-independent manner in colon cancer cells. Meanwhile, sensitivity to chemotherapeutic drugs in colon cancer cells primarily depends on genetic variation [23]. A limitation of this study was that the effect of eupatilin on the Wnt/β-catenin and RAS signaling pathways, which are well characterized in colon cancer was not considered. The Wnt/β-catenin and RAS pathways play important roles in the initial stage and progression of colon cancer tumorigenesis, and mutations in genes belonging to both pathways contribute to the malignancy of colon cancer cells [24]. *APC* mutations in the Wnt/β-catenin pathway and *KRAS* mutations, the major form of RAS, are frequently found in colon cancer. Importantly, mutations in *APC* contribute to 5-FU resistance in colon cancer cells [25]. Furthermore, silencing wild-type and mutant *KRAS* enhances the resistance to 5-FU in colon cancer cell lines [26]. Therefore, the further validation of the effect of eupatilin in colon cancer cells with genetic mutation will lead to conclusive evidence suggesting that eupatilin can exert a therapeutic effect in combination treatment with conventional chemotherapy in colon cancer. In addition, *KRAS* mutations are closely associated with alterations in ROS production and mitochondrial metabolism in colon cancer cells [27]. Furthermore, it has been reported that APC-induced apoptosis in colon cancer cells is dependent on mitochondrial metabolism, and ROS generation also suggests that the mitochondrial-mediated apoptosis of eupatilin, verified in this study, may be involved in the major pathway of colon cancer tumorigenesis [28].

Previous studies have also frequently reported that eupatilin regulates the cell cycle and signaling pathways related to cell proliferation and growth. For instance, eupatilin inhibits endometrial cancer cell growth by inducing G2/M phase cell cycle arrest and regulating ERK and AKT phosphorylation [13]. Eupatilin also suppresses proliferation, migration, and invasion, and it induces cell cycle arrest at the G1/S phase in glioma cells [11,29]. In esophageal cancer cells, eupatilin suppresses cell growth by inhibiting AKT and ERK phosphorylation, as well as by inducing G1 phase arrest [10]. In renal cancer cells, eupatilin activates MAPKs, including ERK1/2, P38, and JNK, and it inhibits the PI3K/AKT pathway [30]. Similarly, as seen in this study, eupatilin inhibits the PI3K/AKT pathway and activates the MAPK pathway in colon cancer cells. However, further research is required to determine whether eupatilin directly affects colon cancer cell survival and growth by regulating this signaling pathway. Moreover, the phosphorylation of AKT plays an important role in the nuclear translocation and transcriptional activity of β-catenin, which is dependent on *APC* mutation [31]. Therefore, further research is needed to determine whether signaling pathways regulated by eupatilin may be involved in therapeutic effects in genetically mutated colon cancer cells.

Our previous study confirmed that eupatilin induces apoptosis in epithelial ovarian cancer cells by regulating the ER–mitochondria axis [14]. The ER is responsible for various physiological processes, including protein synthesis, protein folding, and Ca^2+^ storage. The ER is involved in apoptosis by directly binding to mitochondria and exchanging Ca^2+^ [32]. Many natural products that are effective anticancer adjuvants target mitochondrial dysfunction-associated ER stress [33]. Under ER stress conditions, GRP78 is released from ER transmembrane signal transducers, including IRE1 and PERK with eIF2α as a downstream protein, which activates the unfolded protein response signaling pathway [34]. IRE1 activates JNK, inducing the intrinsic apoptosis pathway [35]. Activated JNK promotes ROS production and triggers mitochondrial dysfunction by inhibiting complex Ⅰ or mitochondrial enzymes [36]. Additionally, IRE1 can induce mitochondrial apoptosis by binding to BAX and BAK proteins. Furthermore, sustained IRE1 induction may be associated with ERK activation, while ERK inhibition prevents IRE1 in cancer cells [37]. However, it is unclear whether eupatilin-regulated ER stress proteins in colon cancer cells are directly related to mitochondrial apoptosis or the activation of JNK and ERK proteins. Further studies are required in this aspect.

Autophagy is a conserved physiological process involving cellular component degradation to maintain cell homeostasis and survival under stress conditions [38]. In cancer cells, autophagy can promote or inhibit tumors [39]. The pro-survival autophagic process contributes to the growth and aggressiveness of cancer cells under stressful conditions [40]. On the other hand, autophagic cell death induction enhances the therapeutic effect on cancer cells [41]. Autophagy and apoptosis mechanisms have a complex relationship [42]. ATG5 and P62 play crucial roles in the initiation and progression of autophagosome formation [43]. Additionally, the LC3-phosphatidylethanolamine conjugate LC3BII is recruited to autophagosomal membranes and utilized as an autophagy marker. For the first time, we have shown that eupatilin can regulate the expression of the autophagy-related proteins ATG5, P62, and LC3B.

Cancer cell invasiveness is essential for tumor formation and metastasis. Since colon cancer frequently metastasizes, suppressing invasion and migration (which are the underlying physiological mechanisms of metastasis), targeting them is crucial to prevent advanced colon cancer. Just as eupatilin inhibited the invasion and migration of colon cancer cells in this study, previous studies have shown similar effects on other cancer types. For instance, eupatilin inhibits the migration and invasion of human hepatocarcinoma [44]. Eupatilin also suppresses the invasive potential of gastric cancer cells by inhibiting the expression of metalloproteinases [45].

## 5. Conclusions

This study is the first to reveal eupatilin’s apoptotic effect on colon cancer cells and its physiological mechanisms. In this study, eupatilin induced apoptosis mediated by oxidative stress and mitochondrial damage in colon cancer cells. The results that eupatilin regulates a variety of signaling mechanisms suggest that further studies on the relationship between the proteins and physiological properties that eupatilin regulates are required. Moreover, combining eupatilin with standard chemotherapy had synergistic effects, suggesting that eupatilin is a potential therapeutic adjuvant for colon cancer. However, this study did not focus on the role of eupatilin in regulating the Wnt/β-catenin and RAS pathways that are majorly involved in the progression of colorectal cancer. In a future study, it will be necessary to verify the effect of eupatilin in colon cancer cells with mutations in genes such as *APC*, *β-catenin*, and *KRAS*.

## Figures and Tables

**Table 1:** Primary antibodies used in Western blot.

Primary Antibodies	Dilution	Supplier	Catalog Number
BCL-xL	1:1000	Cell Signaling Technology	2764
BAK	1:1000	Cell Signaling Technology	12105
Cytochrome C	1:1000	Cell Signaling Technology	11940
TUBA	1:2000	Santa Cruz	sc-5286
Phosphor-AKT (Ser^473^)	1:1000	Cell Signaling Technology	4060
AKT	1:1000	Cell Signaling Technology	9272
Phosphor-P70S6K (Thr^421^/Ser^424^)	1:1000	Cell Signaling Technology	9204
P70S6K	1:1000	Cell Signaling Technology	2708
Phosphor-S6 (Ser^235^/Ser^236^)	1:1000	Cell Signaling Technology	2211
S6	1:1000	Cell Signaling Technology	2217
Phosphor-ERK1/2 (Thr^202^/Tyr^204^)	1:1000	Cell Signaling Technology	9101
ERK1/2	1:1000	Cell Signaling Technology	4695
Phosphor-P90RSK (Ser^573^)	1:1000	Cell Signaling Technology	9346
P90RSK	1:1000	Cell Signaling Technology	9335
Phosphor-P38 (Thr^180^/Tyr^182^)	1:1000	Cell Signaling Technology	4511
P38	1:1000	Cell Signaling Technology	9212
Phosphor-JNK (Thr^183^/Tyr^185^)	1:1000	Cell Signaling Technology	4668
JNK	1:1000	Cell Signaling Technology	9252
IRE1α	1:1000	Cell Signaling Technology	3294
Phosphor-eIF2α (Ser^51^)	1:1000	Cell Signaling Technology	3398
eIF2α	1:1000	Cell Signaling Technology	5324
GRP78	1:1000	Santa Cruz	sc-13968
ATG5	1:1000	Cell Signaling Technology	12994
Phosphor-P62 (Ser^349^)	1:1000	Cell Signaling Technology	16177
P62	1:1000	Cell Signaling Technology	88588
LC3B	1:1000	Cell Signaling Technology	3868
TYMS	1:1000	Cell Signaling Technology	9045

## Data Availability

Data are contained within the article.
